# Supplementary material for: An open-source framework for large-scale, flexible evaluation of biomedical text mining systems
Source: J Biomed Discov Collab. 2008 Jan 29;3:1. doi: 10.1186/1747-5333-3-1 (PMC2276192; doi:10.1186/1747-5333-3-1)
Supplement: Additional file 1 — Evaluation platform source code. The additional file contains the software evaluation framework discussed in this paper. The most current release can be downloaded from SourceForge.net [1]. See the accompanying README file for instructions on installation and use. Also included in the distribution is a collection of UIMA wrappers for some commonly used BioNLP tools and annotated corpora. [file 1747-5333-3-1-S1.zip › bionlp_uima_repository-1.0.1/docs/api/edu/uchsc/ccp/util/external/stanfordparser/TypedDependencyCls.html]

TypedDependencyCls (Center for Computational Pharmacology Util/UIMA API)


|  |  |  |
| --- | --- | --- |
| **Overview** \| **Package** \| **Class** \| **Tree** \| **Deprecated** \| **Index** \| **Help** | |  |
| **PREV CLASS**   **NEXT CLASS** | **FRAMES**    **NO FRAMES**     **All Classes** |  |
| SUMMARY: NESTED \| FIELD \| CONSTR \| METHOD | DETAIL: FIELD \| CONSTR \| METHOD |  |


---


## edu.uchsc.ccp.util.external.stanfordparser Class TypedDependencyCls

```
java.lang.Object
  edu.uchsc.ccp.util.external.stanfordparser.TypedDependencyCls
```

---

``` public class TypedDependencyCls extends Object ```

---

**Constructor Summary**

`TypedDependencyCls(int governorToken,
int dependentToken,
String relation)`


| **Method Summary** | |
| --- | --- |
| `int` | `getDependentTokenNum()` |
| `int` | `getGovernerTokenNum()` |
| `String` | `getRelation()` |
| `void` | `setDependentTokenNum(int dependentTokenNum)` |
| `void` | `setGovernerTokenNum(int governorTokenNum)` |
| `void` | `setRelation(String relation)` |
| `String` | `toString()` |

**Methods inherited from class java.lang.Object**

`clone, equals, finalize, getClass, hashCode, notify, notifyAll, wait, wait, wait`

**Constructor Detail**

### TypedDependencyCls

```
public TypedDependencyCls(int governorToken,
                          int dependentToken,
                          String relation)
```


**Method Detail**

### getDependentTokenNum

```
public int getDependentTokenNum()
```

---


### setDependentTokenNum

```
public void setDependentTokenNum(int dependentTokenNum)
```

---


### getGovernerTokenNum

```
public int getGovernerTokenNum()
```

---


### setGovernerTokenNum

```
public void setGovernerTokenNum(int governorTokenNum)
```

---


### getRelation

```
public String getRelation()
```

---


### setRelation

```
public void setRelation(String relation)
```

---


### toString

```
public String toString()
```

:   **Overrides:**: `toString` in class `Object`


---


|  |  |  |
| --- | --- | --- |
| **Overview** \| **Package** \| **Class** \| **Tree** \| **Deprecated** \| **Index** \| **Help** | |  |
| **PREV CLASS**   **NEXT CLASS** | **FRAMES**    **NO FRAMES**     **All Classes** |  |
| SUMMARY: NESTED \| FIELD \| CONSTR \| METHOD | DETAIL: FIELD \| CONSTR \| METHOD |  |


---
